# Involvement of dysadherin and E-cadherin in the development of testicular tumours

**DOI:** 10.1038/sj.bjc.6602880

**Published:** 2005-12-06

**Authors:** A Batistatou, C D Scopa, P Ravazoula, Y Nakanishi, D Peschos, N J Agnantis, S Hirohashi, K A Charalabopoulos

**Affiliations:** 1 Department of Pathology, University of Ioannina Medical School, Ioannina, Greece; 2 Department of Pathology, University of Patras Medical School, Patras, Greece; 3 Pathology Division, National Cancer Center Research Institute, Tokyo, Japan; 4 Department of Forensic Sciences, University of Ioannina Medical School, Ioannina, Greece; 5 Department of Physiology, Clinical Unit, University of Ioannina Medical School, Ioannina, Greece

**Keywords:** dysadherin, E-cadherin, seminoma, embryonal carcinoma, lymphoma, testicular tumours

## Abstract

Testicular neoplasms are comprised of a variety of histologically different forms, and their pathogenesis has not been elucidated. Dysadherin is a recently described cell membrane glycoprotein, which has an anticell–cell adhesion function and downregulates E-cadherin. In this study, we examined immunohistochemically the expression of E-cadherin and dysadherin in 120 testicular neoplasms (37 seminomas-26 classic, five spermatocytic and six anaplastic-, 45 embryonal carcinomas, 10 mixed germ cell tumours, two yolk sac tumours, 10 mature and eight immature teratomas and eight non-Hodgkin B-cell lymphomas), clinical stage I. The intensity, the expression pattern and the percentage of neoplastic cell staining was recorded and correlated with the histologic type and vascular/lymphatic invasion. Dysadherin was not expressed in non-neoplastic germ cells, neither in CIS/ITGCNU, but it was highly expressed in all types of germ cell tumours, that demonstrated either embryonic phenotype or somatic differentiation, in most terminally differentiated neoplasms, and in all lymphomas. Dysadherin expression did not correlate with vascular invasion. Increased dysadherin expression was correlated with aberrant E-cadherin expression in most tumours. In 17% of embryonal carcinomas colocalisation of dysadherin and membranous E-cadherin staining was noted. This is the first report on dysadherin expression and its association with E-cadherin in testicular tumours. Since dysadherin is not normally expressed in non-neoplastic testis, it is conceivable that it plays a role in the neoplastic transformation of germ cells. In testicular tumours, as in other neoplasms, dysadherin downregulates E-cadherin expression, at least in part.

Testicular germ cell tumours (TGCTs) are the most common malignancy in young males, with peak incidence between 20 and 40 years of age (Adami *et al*, 1994). They arise from the germinal epithelium of the seminiferous tubules and are comprised by many histologically different types. Based on their presumed histogenesis and their line of differentiation, a practical classification system for clinical purposes has been adopted (Mostofi and Sobin, 1997; Rosai, 2004). Thus, they are divided into two major categories: seminomas and nonseminomatous germ cell tumours (NSGCTs). According to the WHO classification in the first category belong the classic seminoma, the spermatocytic seminoma and also anaplastic seminomas. In the latter, the major types are the embryonal carcinoma, yolk sac tumour, choriocarcinoma, and teratomas (mature and immature). There are also mixed germ cell tumours that are comprised of various percentages of both seminomas and NSGCTs. It is believed that both seminomas and NSGCTs originate from a common precursor, carcinoma *in situ*/intratubular germ cell neoplasia unclassified (CIS/ITGCNU). Testicular non-Hodgkin lymphoma is an uncommon neoplasm that affects mostly elderly men, accounting for 25–50% of the testicular tumours in men above 50 years of age. The pathogenesis of both TGCTs and testicular lymphomas remains poorly understood.

The cadherin family is a group of Ca^2+^-dependent cell–cell adhesion molecules, which are essential for the induction and maintenance of tissue structures (Takeichi, 1991; Nagar *et al*, 1996) E-cadherin is present in most epithelial cells and plays an important role in the organisation of the epithelial junctional complex. Reduction/loss of E-cadherin has been associated with the development and progression of human carcinomas, by contributing to tumour invasion and metastases (Charalabopoulos *et al*, 2002; Hirohashi and Kanai, 2003). Abnormal E-cadherin expression (heterogeneous, cytoplasmic, or absent) has been detected immunohistochemically in a variety of poorly differentiated, invasive and metastatic carcinomas, such as gastric adenocarcinoma, lobular breast carcinoma, colorectal tumours, prostate adenocarcinoma, pancreatic, and bladder cancer (Charalabopoulos *et al*, 2002, 2004; Chang *et al*, 2002; Hirohashi and Kanai, 2003; Zhang *et al*, 2004; Massarelli *et al*, 2005). Regarding testicular neoplasms, there are less than a handful of studies, with conflicting results (Heidenreich *et al*, 1998; Saito *et al*, 2000; Honecker *et al*, 2004).

Recently, a part of our collaborative team has reported the cloning and characterisation of dysadherin (HSPCii3, IWU-1, KCT1, PIC), a cell membrane glycoprotein, which has an anticell–cell adhesion function and downregulates E-cadherin, in a post-transcriptional manner (Ino *et al*, 2002; Tsuiji *et al*, 2003). So far reports on the function and expression of dysadherin in tumour samples are limited. This novel cancer-associated protein has been detected in gastric, colorectal, pancreatic, esophageal, thyroid tongue, and cervical carcinomas as well as in malignant melanoma and associated with tumour aggressiveness (Aoki *et al*, 2003; Sato *et al*, 2003; Shimamura *et al*, 2003, 2004; Nakanishi *et al*, 2004; Shimada *et al*, 2004a, 2004b; Wu *et al*, 2004; Nishizawa *et al*, 2005). There are no reports on the expression of dysadherin in testicular neoplasms.

The aim of the present study was to examine the expression of dysadherin in testicular GCTs and testicular lymphomas, to associate it with the expression of E-cadherin; and to investigate whether there is correlation with venous/lymphatic invasion.

## MATERIALS AND METHODS

In all, 120 formalin-fixed, paraffin-embedded archival tissue blocks of testicular neoplasms were included in the current study. The material consisted of 37 seminomas (26 classic, five spermatocytic, and six anaplastic), 45 embryonal carcinomas, 10 mixed germ cell tumours, two yolk sac tumours, 10 mature, and eight immature teratomas and eight non-Hodgkin B-cell lymphomas. They corresponded to an equal number of patients (mean age 32.9 years, range 17–78) with clinical stage I testicular neoplasms, as defined by negative radiographic findings of chest, abdomen, and pelvis as well as decrease of the relevant tumour markers after radical orchiectomy (Heidenreich *et al*, 1998). In seven seminomas and nine embryonal carcinomas preinvasive CIS/ITGCNU was noted at the periphery of the invasive tumour. The most representative block from each tumour and in cases of mixed tumours, the block containing all cell types was selected. A section from each block was stained with Haematoxylin and Eosin and examined by two experienced pathologists (AB and CS) for vascular invasion. Lymphatic invasion was recognised when tumour cells either adhered to a vessel wall of usually irregular shape or filled a space lined by endothelial cells without red blood cells, while venous invasion was noted when the vessel wall was thicker and the space contained red blood cells. For statistical purposes both lymphatic and venous invasion were recorded as vascular invasion (VI), as previously reported (Heidenreich *et al*, 1998).

### Immunohistochemistry

We performed immunostaining on formalin-fixed, paraffin-embedded tissue sections using the EnVision System (DAKO), and the monoclonal antibodies: NCC-M53 against dysadherin and E-cadherin (CM170B, Biocare Medical). Briefly, 4 *μ*m-thick tissue sections were deparaffinised in xylene; rehydrated through graded concentrations of alcohol and heated in a microwave oven for two cycles of 15 min each at 300 W, in citrate buffer, for antigen retrieval. Endogenous peroxidase activity was blocked with H_2_O_2_ solution in methanol (0.01 M), for 30 min. After washing with PBS for 5 min, the primary antibodies NCC-M53 (dilution 1 : 1000) and CM170B (dilution 1 : 50) were applied for incubation (30 min at room temperature and overnight at 4°C, respectively). Then the slides were washed for 10 min with PBS and were visualised with the EnVision system using diaminobezidine tetrahydrochloride as a chromogen. Finally, all sections were counterstained with haematoxylin.

### Evaluation of the staining

Two pathologists (AB and CS) without knowledge of the clinical data performed independently semiquantitative evaluation of the staining. There was a high level of conformity, 98.3% for dysadherin and 97.5% for E-cadherin. Where disagreement arose, slides were reviewed together and a consensus view was obtained. For each sample, at least 1000 neoplastic cells were counted, and the percentage of the cancer cells with positive membranous immunostaining as well as the staining intensity were recorded. Staining for both antibodies was graded as 0, if no cells were stained, 1+ if 1–25% of cells were stained, 2+ if 26–49% of cells were stained, 3+ if 50–74% of cells were stained, and 4+ if >75% of cells were stained. For the purposes of statistical analysis, samples that showed weak intensity and/or low frequency of expression (<50%) were grouped together as ‘Low dysadherin or ‘Low E-cadherin’, as previously described (Aoki *et al*, 2003). Staining for E-cadherin was more heterogeneous, since in many cases only granular cytoplasmic staining was noted, while in others a weak incomplete membranous staining was observed. Such staining patterns were considered aberrant.

### Statistical analysis

Analyses were conducted in SPSS software version 11.0 (SPSS Inc., Chicago, IL, USA). For comparisons between antibodies’ expression with clinicopathological variables, we used the chi-square (*χ*^2^) test. All differences were considered statistically significant if *P*<0.05. *P*-values are two-tailed.

## RESULTS

### Dysadherin expression

**Figure 1:**
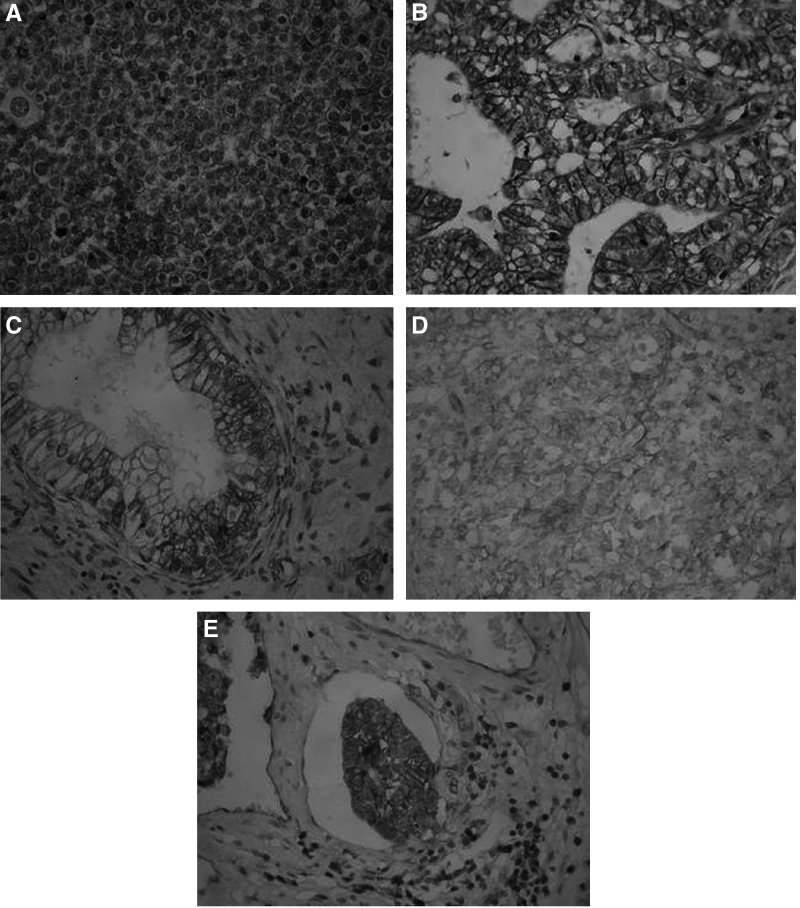
High membranous dysadherin expression in (**A**) classic seminoma, (**B**) embryonal carcinoma, (**C**) epithelial component of mature teratoma, (**D**) non-Hodgkin B-cell lymphoma, and (**E**) neoplastic embolus of embryonal carcinoma.

Dysadherin immunostaining was mostly observed in the membranes of the neoplastic cells and it was homogenous throughout the neoplasm (Figure 1). No dysadherin expression was detectable in adjacent non-neoplastic testis, in any stage of spermatogenesis, or in CIS/ITGCNU. Positive staining of lymphocytes and endothelial cells was used as an internal positive control. Dysadherin was highly expressed in the majority of seminomas, in all yolk sac tumours and embryonal carcinomas, as well as in the epithelial component of mature teratomas (Figure 1A–C). In immature teratomas glandular formations were stained for dysadherin, but not the hypercellular immature stroma or the neuroepithelium (Table 1). In mixed GCT each component retained the expression characteristics consistent with its microscopic appearance (Table 1). Interestingly, in all lymphomas dyasdherin was highly expressed (Figure 1D).

In 10 of the embryonal carcinomas, vascular invasion was noted at the periphery of tumour. These neoplastic emboli also stained positively for dysadherin, in a manner similar to the primary tumour (Figure 1E).

High dysadherin expression was correlated with specific histologic types (*χ*^2^, *P*<0.05), but not with higher incidence of vascular invasion.

### E-cadherin expression

**Figure 2:**
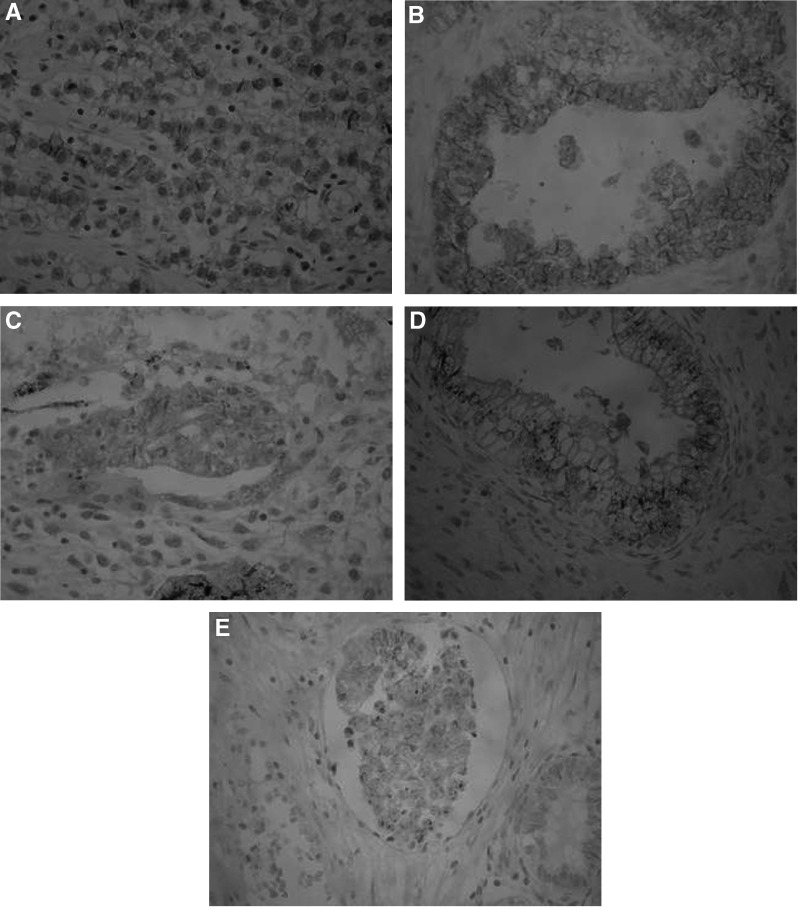
Aberrant E-cadherin expression in (**A**) classic seminoma, (**C**) embryonal carcinoma, (**D**) epithelial component of mature teratoma, and (**E**) neoplastic embolus of embryonal carcinoma. (**B**) High membranous E-cadherin expression at the cell–cell borders of neoplastic embryonal carcinoma cells.

Non-neoplastic germ cells, Sertoli cells, and interstitial cells were all negative for E-cadherin. However, the majority of the tumours showed an heterogeneous intratumoral expression pattern (Figure 2). E-cadherin was not expressed in spermatocytic seminomas (complete absence of staining), while it was aberrantly expressed in 19% of classic seminomas (incomplete membranous staining in >50% of neoplastic cells) and in the majority of anaplastic seminomas (67%) (Figure 2A, Table 1). In the majority of embryonal carcinomas (84%) there was some staining for E-cadherin. Specifically, aberrant cytoplasmic expression of E-cadherin was detected in most of them (67%), while membranous expression in >50% of neoplastic cells was noted in only eight cases (17%) (Figure 2B and C, Table 1). In mixed GCT each component retained the expression characteristics consistent with its microscopic appearance (Table 1). Both yolk sac tumours examined demonstrated aberrant (cytoplasmic) E-cadherin expression. In the epithelial component of mature teratomas aberrant (cytoplasmic) staining for E-cadherin was noted (Figure 2D). In immature teratomas glandular formations were negative for E-cadherin, as well as all other immature tissues recognised. As expected all lymphomas were completely negative for E-cadherin.

Of all the tumours examined colocalisation of dysadherin and membranous E-cadherin staining in >50% of neoplastic cells was noted only in 17% of embryonal carcinomas (judged by comparing tumour areas in adjacent slides). In all other cases, where E-cadherin staining was noted, intense membranous staining for dysadherin was associated with weak/moderate granular cytoplasmic (aberrant) staining for E-cadherin. There was no tumour with high expression of E-cadherin and low expression of dysadherin. There was a reverse association between increased dysadherin expression and decreased/aberrant E-cadherin expression (*χ*^2^, *P*<0.05).

We did not observe a significant association between decreased E-cadherin expression and higher incidence of vascular invasion (*χ*^2^ *P*>0.05). In all 10 cases of embryonal carcinomas where vascular invasion was noted, the neoplastic emboli exhibited E-cadherin staining comparable to that of the primary tumour (Figure 2E).

## DISCUSSION

Since the frequency of testicular neoplasms, which affect primarily young males, has increased in developed countries the search for new molecules possibly involved in their pathogenesis and progression is of high importance (Heidenreich *et al*, 1998; Delgado *et al*, 2000; Aubry *et al*, 2001). Embryonal carcinoma is composed of primitive carcinoma-like cells with minimal or no signs of differentiation. In teratomas the differentiation is towards structures of the embryo proper, usually a combination of endodermic, mesodermic, and ectodermic tissues. Yolk sac tumours are directed towards the formation of extraembryonic endoderm, and mesoderm. Traditionally seminomas have been regarded as ‘end point’ neoplasms incapable of further differentiation in any of the directions of the NSGCTs. However, the presence of anaplastic seminoma is an indicator that there exists a testicular tumour situated between classic seminoma and embryonal carcinoma, and may represent the link between them (Mostofi and Sobin, 1997; Rosai, 2004).

In our study, the expression of dysadherin appears to be high in all types of germ cell tumours, which demonstrate either embryonic phenotype or somatic differentiation as well as in most terminally differentiated neoplasms, that is seminomas. Since dysadherin is not normally expressed in non-neoplastic germ cells, it is conceivable that it is a molecule that plays a pivotal role in the neoplastic transformation of germ cells. Furthermore, it is intriguing that dysadherin in not expressed in CIS/ITGCNU, suggesting that it is a key protein involved in the acquirement of the invasive phenotype, both on seminomas and in NSGCTs. However, dysadherin expression did not correlate with vascular invasion. Since this is the first study on dysadherin expression in testicular neoplasms, it is too early to speculate about the role of this protein in the pathogenesis of these tumours. This is also the first report on dysadherin expression in lymphomas. The latter is not surprising, given that dysadherin is normally expressed in lymphocytes and the fact that lymphomas are well known for the lack of cohesiveness between neoplastic cells. Further studies in lymphomas developing in lymph nodes are needed in order to further examine the role of dysadherin in this large category of neoplasms.

Until now, dysadherin expression has been studied in colorectal, pancreatic, gastric, esophageal, tongue and thyroid carcinomas, as well as in cervical squamous cell carcinomas and cutaneous malignant melanomas (Aoki *et al*, 2003; Sato *et al*, 2003; Shimamura *et al*, 2003, 2004; Nakanishi *et al*, 2004; Shimada *et al*, 2004a, 2004b; Wu *et al*, 2004; Nishizawa *et al*, 2005). In all these cancer types, a general phenomenon is that dysadherin expression seems to reflect tumour aggressiveness, being furthermore a marker of poor prognosis when considered alone or/and in combination with downregulation of E-cadherin. A process involving increased dysadherin expression may lead to an adverse clinical outcome.

Our results also expand the published information on E-cadherin expression in testicular tumours. Heidenreich *et al* (1998) reported E-cadherin expression in 76.1% of NSGCTs, as well as positive staining in benign testicular tissue. The latter is not confirmed by our study, neither by that of Saito *et al* (2000), who showed that E-cadherin was not expressed in normal germ cells. They further demonstrated that E-cadherin was expressed in 18.8% of seminomas and 62.5% of NSGCTs, mainly on the epithelial component of teratoma cells. Honecker *et al* (2004) demonstrated that E-cadherin was not expressed in CIS/ITGCNU, in seminomas and dysgerminomas, while it was found in the majority of NSGCTs. Our results are in accordance with these studies if we take in account the functional membranous as well as the aberrant cytoplasmic E-cadherin immunostaining. It is not possible to further compare our data with the above-mentioned studies, since a detailed analysis of E-cadherin expression is not provided, and none of the figures shows E-cadherin immunostaining in seminomas or embryonal carcinomas. Nonetheless, it appears that E-cadherin protein is synthesised by the neoplastic cells, but probably not directed properly to the cell membrane, in most cases. A corollary to this hypothesis is the information provided by Kawakami *et al* (2003) who using multipoint methylation analysis have not detected methylation in E-cadherin neither in seminomas nor in NSGCTs, but have shown a consistent aberrant E-cadherin methylation in testicular lymphomas . In agreement with this, none of our eight non-Hodgkin B-cell lymphomas showed any expression of E-cadherin. As reported previously, E-cadherin expression did not correlate with vascular invasion (Heidenreich *et al*, 1998).

Regarding seminomas the differential expression of dysadherin and E-cadherin possibly reflects the unique aspects of their pathobiology. Interestingly, spermatocytic seminoma, which is regarded as a low-grade malignancy, showed increased expression of dysadherin and absent E-cadherin, while anaplastic tumours, that have more aggressive behaviour exhibited concomitant high expression of dysadherin and aberrant E-cadherin, similar to that observed in embryonal carcinomas. In classic seminomas the expression of the two molecules is somewhere in between. These findings lend further support to the hypothesis that anaplastic seminomas are the link between seminomas and embryonal carcinomas.

Increased dysadherin expression was correlated with aberrant E-cadherin expression. E-cadherin is produced by ribosomes on the endoplasmic reticulum, as a precursor molecule which is cleaved at preregion, forming a mature molecule of 120 kDa that migrates intracellularly, taking finally a place in the cytoplasmic membrane and acquiring a functional extracellular carboxy domain. Thus, cytoplasmic distribution of E-cadherin represents an aberrant expression pattern, since in this location E-cadherin is not able to mediate cell–cell adhesion interactions.

As was mentioned herein, colocalisation of dysadherin and membranous (normal/functional) E-cadherin staining in more than 50% of neoplastic cells was noted only in 17% of embryonal carcinomas. This finding suggests that other mechanisms apart from dysadherin regulation are implicated in E-cadherin expression/activity both at transcriptional and post-transcriptional level. Dysadherin may only partly suppress/regulate the activity of E-cadherin. Many other factors may also be involved, such as methylation of the promoter region, gene mutation, involvement of snail, sip-1, and slug transcription factors, ubiquination of E-cadherin, tyrosine phosphorylation of *â*-catenin, as well as catenin abnormalities, such as absence of *α*-catenin. In addition, there may be a threshold level of dysadherin for downregulation of E-cadherin, and downregulation of E-cadherin expression is not the only mechanism by which dysadherin affects the aggressiveness of embryonal testicular tumours.

In conclusion, this is the first report on dysadherin expression and its association with E-cadherin in testicular tumours. Expression patterns in all histological types were described and their possible implication in neoplastic transformation have been discussed. Larger prospective studies, fulfilling many clinicopathological parameters are needed in order to clarify the important issue of testicular carcinogenesis.

## Figures and Tables

**Table 1:** Results of immunostaining, differentiated by histological subtype

		**Dysadherin**		**E-Cadherin**		
**Histology**	**No cases**	**High**	**Low**	**High**	**Low**	**Aberrant**
CIS/ITGCNU	16	0	16 (100%)	0	16 (100%)	0
						
*Seminoma (SE)*						
Classic	26	17 (65%)	9 (35%)	0	21 (81%)	5 (19%)
Spermatocytic	5	5 (100%)	0	0	5 (100%)	0
Anaplastic	6	6 (100%)	0	0	2 (33%)	4 (67%)
						
Yolk sac tumour	2	2 (100%)	0	0	0	2 (100%)
						
*Teratoma (epithelial comp.)*						
Mature	10	10 (100%)	0	0	0	10 (100%)
Immature	8	8 (100%)	0	0	8 (100%)	0
						
*Embryonal Carcinoma (EC)*	45	45 (100%)	0	8 (17%)	7 (16%)	30 (67%)
						
*Mixed Germ Cell tumour*	10					
EC component	10	10 (100%)	0	2 (20%)	2 (20%)	6 (60%)
SE component	10	6 (60%)	4 (40%)	0	9 (90%)	1 (10%)
						
Lymphoma	8	8 (100%)	0	0	8 (100%)	0
